# Microscopic Chain Motion in Polymer Nanocomposites with Dynamically Asymmetric Interphases

**DOI:** 10.1038/srep29326

**Published:** 2016-07-26

**Authors:** Erkan Senses, Antonio Faraone, Pinar Akcora

**Affiliations:** 1 NIST Center for Neutron Research, National Institute of Standards and Technology Gaithersburg, MD 20899-8562, USA; 2 Department of Materials Science and Engineering, University of Maryland, College Park, MD 20742-2115, USA; 3 Department of Chemical Engineering and Materials Science, Stevens Institute of Technology, Castle Point on Hudson, Hoboken, NJ 07030-5942.

## Abstract

Dynamics of the interphase region between matrix and bound polymers on nanoparticles is important to understand the macroscopic rheological properties of nanocomposites. Here, we present neutron scattering investigations on nanocomposites with dynamically asymmetric interphases formed by a high-glass transition temperature polymer, poly(methyl methacrylate), adsorbed on nanoparticles and a low-glass transition temperature miscible matrix, poly(ethylene oxide). By taking advantage of selective isotope labeling of the chains, we studied the role of interfacial polymer on segmental and collective dynamics of the matrix chains from subnanoseconds to 100 nanoseconds. Our results show that the Rouse relaxation remains unchanged in a weakly attractive composite system while the dynamics significantly slows down in a strongly attractive composite. More importantly, the chains disentangle with a remarkable increase of the reptation tube size when the bound polymer is vitreous. The glassy and rubbery states of the bound polymer as temperature changes underpin the macroscopic stiffening of nanocomposites.

Addition of nanoparticles into polymer matrices remarkably changes mechanical^1^,^2^, optical^3^, and electrical properties^4^,^5^ relative to the host polymer. It is known that polymer-surface interactions play a central role in determining dynamics of interfacial polymers^6^,^7^ which controls the macroscopic properties of polymer nanocomposites. Repulsive or attractive polymer-surface interactions often result in enhanced mobility or slower aging as commonly studied with confined polymers in thin films^8^,^9^ or nanopores^10^. In addition to the strength of attraction, the rigidity of the neighboring polymer has significant effect on tuning the glass transition temperature (T_g_) and dynamics of the confined polymer^11^,^12^,^13^. Polymer nanocomposites often involve a confined layer close to the particle surface and a bulk-like matrix with an interphase layer of matrix chains around the fillers^14^,^15^,^16^. Understanding the microscopic dynamics within the interphase layer is the aim of this study and is essential in improving properties of hybrid materials.

It is known that large scale dynamics of a long entangling chain is controlled by the time scale of the segmental motions (Rouse dynamics) and the length scale of the confinement induced by the neighboring chains (reptation tube diameter). Experimental and computational studies have investigated the effect of nanoparticles on the Rouse dynamics and chain entanglements in weakly interacting/repulsive composites^17^,^18^,^19^. A commonly reported result in attractive nanocomposites (*i.e.* composites with attractive interactions between particles and polymer) is the slowing down of the bound chains on nanoparticles^20^,^21^,^22^. The hypothetical glassy bound layer and its percolation were postulated to reinforce nanocomposites^23^. Recent dynamic measurements^15^,^24^, however, showed that the interfacial polymer can be highly mobile with no glassy nature, albeit with reduced segmental relaxations, and that the confined dynamics of the chains can translate into the bulk phase through strong topological interactions (entanglements)^15^. The effect of the state (glassy vs. rubbery) of the interfacial polymer on the macroscopic dynamics remains elusive.

It is difficult to measure the dynamics of highly entangled chains in matrix and interphases when all chains are chemically identical. In our previous work^25^, we showed that 55-nm silica nanoparticles adsorbed with a high-T_g_ polymer, poly(methyl methacrylate) (PMMA), were individually dispersed in a miscible low-T_g_ matrix, poly(ethylene oxide) (PEO). Mechanical relaxations of composites were measured in multiple temperature sweep experiments. Composites with glassy-bound polymer behaved unusually liquid-like at temperatures lower than T_g_ of PMMA with very low viscosity close to that of pure matrix while they behaved highly elastic and reinforced when the PMMA layer was mobile above its T_g_. Due to the negligible enthalpic interaction between PEO and PMMA^26^,^27^, the system can be viewed as a model nanocomposite with weakly attractive polymer-particle interfaces where the interfacial polymer change from glassy to rubbery states upon heating within the same dispersion state. This work is designed to understand the role of nanoparticle-polymer interphases on multi-scale chain relaxations.

We employ quasi-elastic neutron scattering on our nanocomposites with isotopically labeled and dynamically asymmetric bound (PMMA) and matrix (PEO) chains to evaluate the role of interfacial PMMA on the microscopic dynamics of PEO at space-time resolutions relevant to segmental and collective chain motion^28^,^29^. We report a direct experimental observation of a dilation of the reptation tube size (≈25% compared to the bulk) with glassy interfaces. The recovery of the chain confinement at elevated temperatures underlines the importance of interfacial polymer mobility. For polymer nanocomposites and thin films, these results motivate a rethinking about the role of the state of irreversibly adsorbed polymer on microscopic chain dynamics and the resulting macroscopic mechanical properties.

## Results

### Nanoparticle Dispersion and Polymer Conformation

Three samples, namely, PEO, PEO-bare Silica (SiO_2_) and PEO-PMMA adsorbed SiO_2_ were prepared. We used neutron spin-echo^29^, which directly measures the Intermediate Scattering Function (ISF) using the precession of the neutron spin in a magnetic field to obtain the collective single chain PEO dynamics in nanocomposites. 55-nm SiO_2_ nanoparticles were made invisible to neutrons by mixing deuterated and protonated chains for PEO and PMMA at 52/48 and 44/56 ratios, respectively (see Supplementary Table 1 for complete list). To study the Rouse dynamics, we used backscattering using fully hydrogenated PEO and fully deuterated PMMA (see Methods section for details).

**Figure 1:**
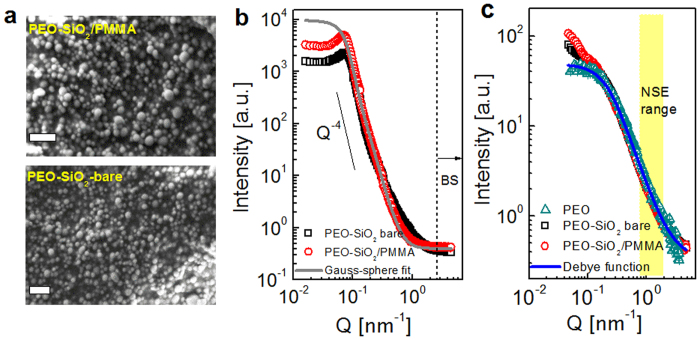
Nanoparticle dispersion and chain conformation in composites. (**a**) Scanning electron micrographs on freeze-fractured surfaces of composites with bare and PMMA-adsorbed particles used in NSE (scale bars: 200 nm) (**b**) SANS of composites with fully protonated PEO and fully deuterated PMMA (backscattering samples) reveals the particle dispersion. Spherical particles with radii of 25 nm and Gaussian dispersity of 0.3 are used to fit the data down to the structure peak at ≈0.0075 Å^−1^. Vertical dashed line and arrow show the Q-range used in backscattering (**c**) SANS profiles of the composites in partially deuterated PEO. The silica particles are made invisible via zero-average contrast matching; scattering reveals the form factor of PEO chains in the matrix. The curve is the prediction from the Debye model.

Good dispersion of particles is essential to investigate the interphase effects in nanocomposites. SEM images of bare and PMMA coated Silica (SiO_2_) filled PEO are shown in Fig. 1a. To visually assess the dispersion quality, we provide the SEMs of composites with more hydrophobic (-CH_3_ terminated) PEO which resulted in severe agglomeration (Supplementary Figure 1). PMMA-PEO systems are known to be miscible up to 500 K with a very weak energetic interaction (Flory-Huggins parameter, χ < −10^−3 ^26,27). The pre-adsorbed PMMA chains (see Supplementary Figure 2) provide steric protection against particle aggregation. We determined face-to-face interparticle distance of ≈29 nm for individually dispersed particles using 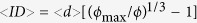 with *ϕ*_max_ = 2/*π* (for maximum random packing) and *φ* = 17.6% (particle volume fraction) and <*d>* = 55 nm (average particle size). The small-angle neutron scattering (SANS) profiles from d-PMMA adsorbed particles in h-PEO (same isotopic composition used in the backscattering) confirms even dispersion (see Fig. 1b). For both bare and PMMA coated particles, a well-defined low-q plateau is achieved suggesting the absence of structures larger than individual particles. *I* ∝ *Q*^−4^ scaling at higher *Q* reveals sharp surfaces of the individually dispersed particles. The SANS profiles are fit to the polydisperse sphere form factor with R = 25 nm and polydispersity index of 0.3 that match the expected size and distribution of the particles. The interference peak at *Q* ≈ 0.075 nm^−1^ corresponds to an average face-to-face interparticle distance (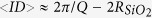) of ≈29 nm and matches with the predictions from the theoretical distance from the random particle distribution. The chain size (*R*_*g*_) of PEO is determined to be 6.85 nm from 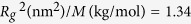
30. Since <*ID*> ≈ 4.2*R*_*g*_, the matrix chains are not under strong geometric confinement and composites consist of both interfacial and bulk chains. Similar dispersion states of bare and PMMA coated particles in PEO matrix allow us to keep the geometric confinement the same for the samples and unequivocally evaluate the dynamic effects of the thin interfacial PMMA layer.

Figure 1c shows the SANS profiles of PEO homopolymer, and composites with bare and PMMA adsorbed particles. The solid curve is the Debye form factor, 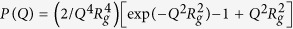, with *R*_*g*_ = 6.85 nm. Slight upturns at low-*Q* is presumably due to the additional coherent contribution from the sharp structure peak of SiO_2_. Lastly, it is important to note that the particle dispersion does not change with temperature^25^.

### Macroscopic Dynamics

**Figure 2:**
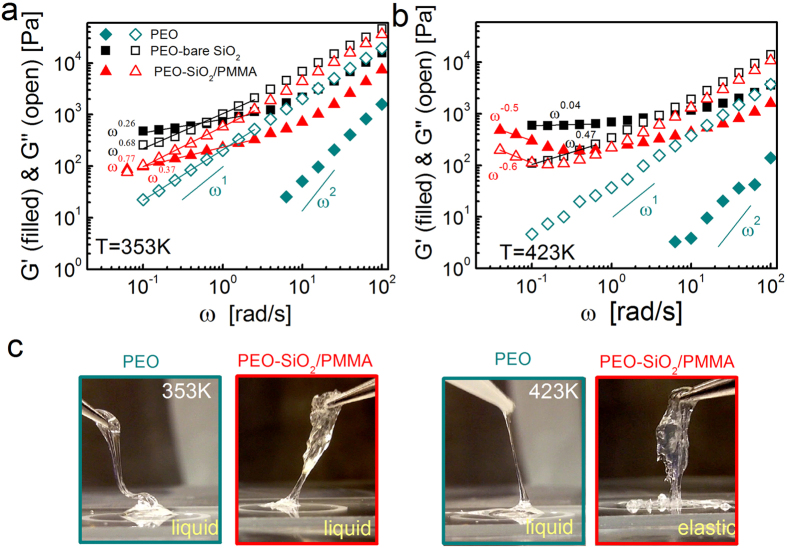
Macroscopic stiffening of PEO composite with temperature. The linear elastic (*G*′, open symbols) and viscous (*G*″, closed symbols) modulus as a function of angular frequency (*ω*) at 353 K (in (**a**) and 423 K (in (**b**)) for PEO melt, and nanocomposites filled with bare and PMMA-adsorbed SiO_2_ at 30 wt%. (**c**) PEO and PEO-SiO_2_/PMMA composite above and below T_g,PMMA_. Particle-free PEO becomes less viscous while PEO-SiO_2_/PMMA becomes elastic at high temperature.

Macroscopic dynamics of composites is studied in melt rheology. Figure 2a,b displays the elastic (*G*′) and viscous (*G*″) moduli at 353 K and 423 K. The frequency sweep response at 353 K clearly shows that both bare and PMMA adsorbing SiO_2_ particles have reinforcing effects relative to PEO. It is interesting to observe that the composite with frozen PMMA chains is significantly less reinforced compared to the composite with bare particles. The cross-over modulus of PMMA-coated particles appears at a lower frequency than the attractive bare-particle composite (Fig. 2a). As seen in the viscoelastic response at high frequency measured at 423 K in Fig. 2b, stiffening in the PMMA-coated particles becomes apparent only at low frequency since PMMA is still glassy at high deformation rates. It is apparent that both composites have different mechanical response at 353 K and 423 K compared to PEO homopolymer (*G*′ ∝ *ω*^2^ and *G*″ ∝ *ω*^1^)^31^. Photographs in Fig. 2c show liquid and solid-like look of the composite at 353 K and 423 K. Particle-free liquid PEO becomes less viscous while PEO-SiO_2_/PMMA turns from viscous liquid to elastic upon heating above *T*_*g*,*PMMA*_. The bare particle composite remains elastic at both temperatures and becomes more deformable at higher temperature (see Supplementary Figure 3 for images). From the macroscopic point of view, bulk rheology clearly suggests that the mobile bound polymer is more desirable for mechanical reinforcement than a glassy layer on particles: a rather counter-intuitive finding.

Terminal relaxation time for PEO homopolymer is estimated from rheology data. Center of mass diffusion of the reptating chains governs the viscous behavior with zero-shear viscosities, 
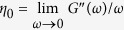
 of 230 Pa-s and 50 Pa-s at 353 K and 423 K, respectively. In the rubbery plateau, zero-shear viscosity is related to the modulus by 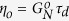, however, we did not observe a plateau regime for 35 kg/mol PEO at these temperatures. We used a higher molecular weight PEO (100 kg/mol) at 348 K (Supplementary Figure 4) to estimate the entanglement relaxation time, 
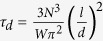
, where *N* is the number of segments, *l* = 0.58 nm is the segmental length^32^, d = 5.3 nm is the tube size^33^ and *W* = 3*k*_*B*_*T*/*l*^2^*ζ* is the elementary Rouse rate with monomeric friction coefficient, *ζ*. Knowing that *τ*_*d*_ ∝ *N*^3.4^, corresponding terminal relaxation time for 35 kg/mol PEO at 348 K was then estimated to be *τ*_*d*_ ≈ 1.3 ms. The characteristic Rouse rate of PEO is found to be 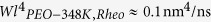, a comparable number to previously reported values^33^,^34^ of *τ*_*d*_ from rheology.

### Microscopic chain dynamics

Next, we examined the relaxation at the microscopic level. Due to the hierarchical structure of polymers, distinct relaxation processes occur at the segmental and chain levels, and control the dynamics at larger scales^35^. The macroscopic motion of a long polymer chain is generally described by two microscopic parameters: (i) the elementary Rouse relaxation time, effective locally at the segmental level and at short times, and (ii) the entanglement spacing, in which chain motion is confined within a reptation tube, with intermediate length/time scales (typically 5 to 10 nm). Thus, the main goal of this section is to clarify how the local segmental dynamics and tube diameter are affected by the mobility of interfacial PMMA.

We measured the intermediate coherent dynamic structure factor at *Q* values from 0.8 nm^−1^ to 2 nm^−1^, corresponding to length scales from ≈3 to 8 nm, using NSE. Both PEO and PMMA were contrast-matched to SiO_2_ to make the particles invisible to neutrons, and the coherent scattering from single PEO chains are obtained from hydrogenated PEO chains in the deuterated matrix. The scattering intensity from interfacial PMMA chains in the composites is too small to significantly contribute to the observed dynamics (see Supporting information). The collective motion of linear short chains or entangled chains at short times is well described by an unrestricted Rouse motion where the chain does not feel the tube confinement. The coherent intermediate scattering function is expressed as


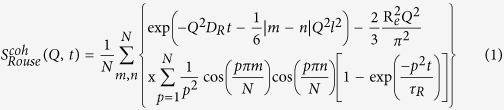


where 
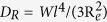
 is the Rouse diffusion constant, *R*_*e*_ = *Nl*^2^ is the chain end-to-end distance, and *τ*_*R*_ = *N*^2^/(*π*^2^*W*) is the Rouse time. 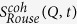 describes the combined effects of center of mass diffusion, static structure factor and relaxations of the internal modes. The only dynamic parameter for the Rouse motion is the elementary Rouse rate, *W*.

For long chains, entanglements dominate the dynamics and the chains feel a confining tube and exhibit a local reptation motion. The collective dynamics within a confining tube is well described by de Gennes’ model^36^ as


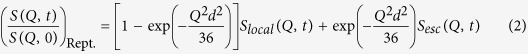



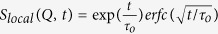
 is the local reptation within the tube with characteristic time-scale *τ*_*o*_ = 36/(*Wl*^4^*Q*^4^). *S*_*esc*_(*Q*, *t*) describes the long-time creeping of the chain out of its original tube on the time scale of *τ*_*R*_; consequently *S*_*esc*_(*Q*, *t*) = 1 for the motions probed by NSE since *t*_*NSE*_ < 10^2^ ns ≪*τ*_*R*_(≈10 *μ*s) in the present work. The term 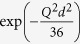 is the cross-sectional form factor of the tube with diameter *d* and determines the long time-plateau level.

Apparently, the collective motion of an entangling chain at intermediate times is determined by two quantities: the Rouse parameter, *Wl*^4^, and the confinement length scale, d. While these can be determined for particle-free linear polymers from their bulk rheology, it is under debate how nanoparticles affect these two parameters in nanocomposites (details in discussion section). We thus first determined effective Rouse parameters by fitting the reptation model (equation 2) to the PEO data with free Rouse parameters (shown as solid curves in Fig. 4a). The tube diameters from the global fittings are determined to be 5.6 ± 0.4 nm and 5.6 ± 0.2 nm at 353 K and 423 K, respectively a. These are close to the previously reported numbers for pure PEO^33^. The effective Rouse rates from NSE are found to be 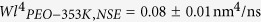 and 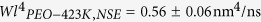.

**Figure 3:**
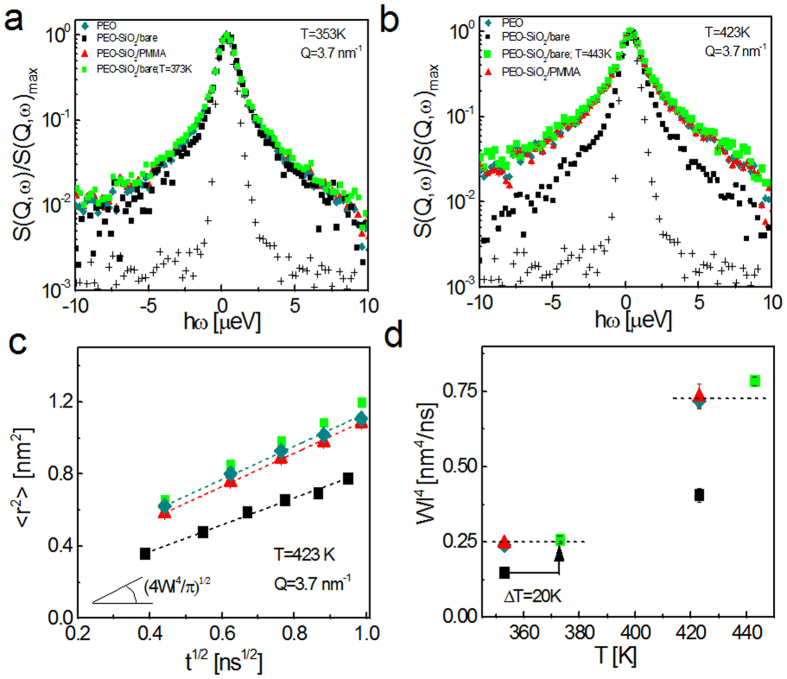
Incoherent dynamic structure factor and mean square displacement of PEO. Dynamic structure factor of PEO and nanocomposites in the frequency domain obtained in backscattering at (**a**) low and **(b**) high temperature. Symbols (+) represent the resolution data for neat PEO taken at ≈10 K. (**c**) Mean-square displacement (MSD) obtained from the inverse-Fourier transformed backscattering data at *Q* = 3.64 1/nm plotted on Rouse scaling (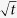). (**d**) Rouse Parameters (Wl^4^) of PEO in particle-free form and nanocomposites reveal unchanged monomeric relaxation in weakly interacting PMMA interfaces and slowing down in the bare system, which requires 20 K higher temperature to reach similar Rouse rates (green squares).

We also calculated the Rouse rates from incoherent backscattering. Figure 3a,b displays the self-dynamic structure factors, 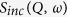, in frequency domain at *Q* = 3.7 nm^−1^ at 423 K and 353 K obtained from the backscattering samples (data for *Q* = 4.7 nm^−1^ in Supplementary Figure 5). The quasi-elastic broadening with respect to the resolution is primarily due to the motions of protons (see Methods section for details) and inversely related to the relaxation time. Remarkably, the broadening is identical for PEO in neat form and in the composites with PMMA adsorbed particles regardless of temperature, implying unchanged segmental dynamics with weakly interacting PMMA interfaces. PEO relaxation appears to be slowing down in attractive bare SiO_2_ composites.

To further quantify, we calculated the mean square displacements (MSD) by Fourier transformation of the dynamic structure factor into time domain using the Gaussian approximation, 
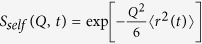
. The spatial range was chosen such that the *Q* range is sufficiently low to obtain segmental level information. With *l* = 0.58 nm, the highest limit for *Q* to observe the Rouse motion is estimated to be *Q* ≈ 10 nm^−1^ which is above the *Q*-range we present here (3.64 and 4.67 nm^−1^). An earlier work from Richter *et al*.^34^ also showed that the Rouse motion of PEO is captured in backscattering up to *Q* = 6 nm^−1^. For linear polymers, MSD is directly related to the elementary Rouse rate, *W*, by 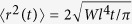. Figure 3c shows a representative MSD at 423 K and at *Q* = 3.7 nm^−1^ where the x-axis is the Rouse scaled time, *t*^*1/2*^. The corresponding Rouse parameters (Fig. 3c) for neat PEO and composite samples reveal that the Rouse rate remains unchanged with weakly attractive PMMA interfaces while highly attractive bare particles cause ≈40% slower dynamics. The reduction of the Rouse rates is likely due to physical adsorption of PEO on attractive bare particles, which greatly extends (beyond the experimental time-scale ≈1 ns) the relaxation of segments that are in direct contact with the surface. The immobilized layer in fact causes an elastic incoherent background^37^ in the self-intermediate scattering function. The value we obtain from the bare composite represents an average behavior with much slower rates close to the particle-polymer interface. Nonetheless, the main conclusion obtained from the incoherent backscattering experiments on the self-dynamics is that the PEO segmental dynamics remains unaffected in composites with PMMA coated particles. Note that the PEO in bare-particle composites requires 20 K higher temperature to reach dynamics similar to that of the bulk form (see Fig. 3d and Supplementary Table 2). An approximately 70% slower Rouse rate estimated from NSE with respect to backscattering at 353 K is at least in part due to the trace amount of PMMA in the samples (<6% PMMA in PEO), which on a larger scale increases the monomeric friction of PEO as also reported elsewhere^38^,^39^. This effect is expected to be larger at low temperature where PMMA is glassy; the difference seems to vanish (within the uncertainties) at 423 K when PMMA is mobile. Another possibility for the slow Rouse rates in NSE may be the incoherent scattering from the hydrogenated chains. The samples investigated by NSE contain a high fraction (49%) of protonated polymer, in order to match the scattering of nanoparticles. The same fraction of protonated polymer is present in the bulk PEO sample measured as reference. Such high amount of protons produces large incoherent scattered intensity. The incoherent scattering function reflecting chain dynamics is expected to be faster than the collective function, and contributes negatively (multiplied by −1/3) to the NSE signal. This leads to effectively slower decays of the NSE signal as compared to the purely coherent contribution. This may also cause the discrepancies between the values deduced for the Rouse rate from NSE and backscattering measurements.

**Figure 4:**
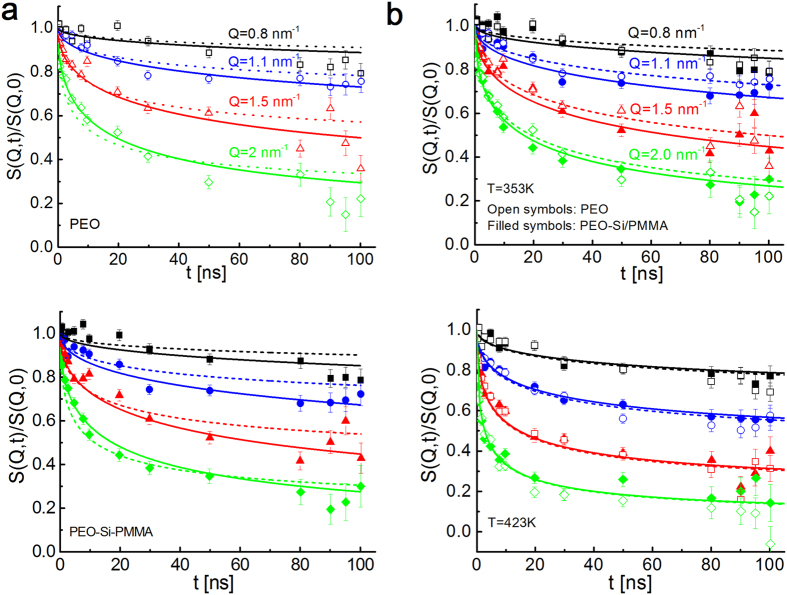
Neutron spin-echo and fitting results. (**a**) PEO and PEO-SiO_2_/PMMA composite at 353 K. Dashed and solid curves are global fittings to de-Gennes reptation model using Rouse rates (*Wl*^4^) obtained from backscattering and effective rates determined from NSE. (**b**) Comparison of the NSE data and corresponding reptation model fits of particle-free PEO and PEO in composite with PMMA-coated particles at T < *T*_*g*,*PMMA*_ and T > *T*_*g*,*PMMA*_.

While the reduced Rouse rates may be responsible for higher reinforcement in attractive bare particle composites (see Fig. 2a), the thermal stiffening with PMMA adsorbed particles may not be explained by the slowing segmental dynamics because the Rouse rates in these composites remain unchanged with respect to pure PEO. This raises an interesting question of whether the self-confinement of the entangling PEO changes when the interfacial polymer vitrifies. We then looked at the collective dynamics to estimate the length-scale of the tube confinement. The Rouse model, equation 1, fails to explain the long-time plateau as it is valid for short non-entangling chains (see Supplementary Figure 6 for fitting to PEO data at 423 K). Dashed curves in Fig. 4a are global fittings of the de-Gennes formulation, equation 2, to the NSE results for PEO and PEO-PMMA-adsorbed SiO_2_ composites at 353 K with *Wl*^4^ obtained from backscattering. Apparently the model deviates from the experimental results at both short and long times by predicting faster decay at short times and a higher confinement plateau (see Supplementary Figure 7 for the deviations at short times). The tube diameter of PEO obtained from this fitting was 3.7 ± 0.1 nm. This value shows a clear deviation from the numbers (≈5.3 nm^33^) reported in previous works for neat PEO (ref. 34 and references therein).

We followed the protocol used in previous studies by Niedzwiedz *et al*.^33^. The reptation model was fit to the composite data using the coherent Rouse rates of PEO for t > 20 ns to obtain corresponding tube sizes. The curves were then extrapolated to t < 20 ns. Figure 4b compares PEO and PEO-SiO_2_/PMMA composite results at 353 K and 423 K. Note that the short-time behavior of PEO in bulk and composites are almost identical at both temperatures. Effect of PMMA mobility manifests at longer times. At 353 K, PEO chains experience less confinement when PMMA is glassy. The corresponding tube diameter is 6.78 ± 0.3 nm; ≈25% larger than its bulk value. Remarkably, the tube size recovers when PMMA is mobile at 423 K and the PEO relaxation is identical to its particle-free form. Since the Rouse rates of the composites are identical to that of pure PEO, disentanglement and entanglement of the matrix chains by the glassy and mobile PMMA interfaces is responsible for a softening-stiffening transition with temperature as measured from bulk rheology (Fig. 2).

**Figure 5:**
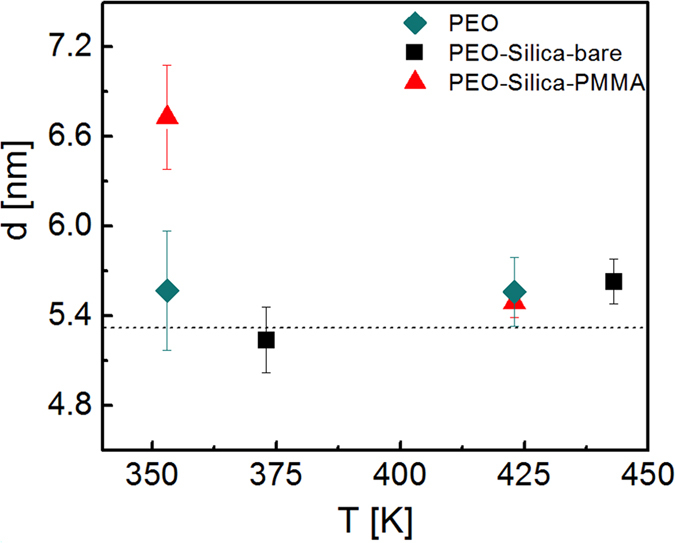
Reptation tube diameter of PEO. The tube size obtained from NSE for the PEO homopolymer and PEO in the composites. The dashed line shows the previously reported value for PEO^33^.

In the absence of a PMMA layer, the attractive bare particles did not modify the tube-confinement of the matrix chains as in the composite with PMMA adsorbed particles. We perfomed NSE measurements for this composite at 20 K higher temperatures (373 K and 443 K) where the PEO segmental relaxation rates (*Wl*^4^) become comparable to those in the neat PEO and the composites with PMMA bound particles (see Fig. 3d). Fittings are displayed in Supplementary Figure 8. The reptation tube diameters from the collective dynamic measurements are presented in Fig. 5.

## Discussion

The PEO-bare particle composite is an attractive system where PEO chains directly interact with SiO_2_ forming a surface bound layer^40^. Surface-pinning of PEO retards the diffusive motion of chains through the entanglements. Our work experimentally shows that the attractive particles indeed slow down the Rouse rate, in agreement with simulations predicting slower Rouse rates in attractive nanocomposites^41^,^42^,^43^. A recent work rheological and dielectric spectroscopy on P2VP nanocomposites with attractive well-dispersed silica nanoparticles inferred increased friction in the composites^43^. This is contrary to the work by Glomann *et al*.^44^,^45^, where Rouse dynamics of short PEG chains was found to be unaffected by highly attractive silica particles. Despite the reduced Rouse rate, we did not observe any significant effect of attractive bare particles on the entanglement tube diameter which is contrary to the observation by Martin *et al*.^46^. However, in that work the confinement was probably the dominating factor since the polymer was filled within pores much smaller than individual chain size. Overall, we conjecture that the well-dispersed attractive nanoparticles, with reduced segmental relaxation and unaffected tube size, would result in a monotonic change of the mechanical properties, such as elastic modulus and viscosity.

A recent MD simulation by Kalathi *et al*.^18^ reported reduced entanglements in the presence of weakly interacting well-dispersed particles when particle size is larger than tube diameter. It was shown that the Rouse rates were affected only at very high particle concentrations at chain sizes comparable to interparticle spacing^18^,^47^. Schneider *et al*.^48^ showed on PEP-Silica that Rouse dynamics remained unchanged in composites and the apparent tube diameter decreased at high particle loadings due to geometric confinement. Li *et al*.^17^ found that the proposed geometric confinement was negligible below the percolation threshold of the spherical particles (31 vol%) and chains disentangle upon filling with repulsive particles, which causes a significant reduction of bulk viscosity as observed in athermal nanoparticle-polymer mixtures^49^. In a recent study^50^, Arbe *et al*. reported significant enlargement of PEO (95 kg/mol) tube size when blended with cross-linked PMMA nanoparticles. This composite system is different from conventional inorganic nanoparticle-polymer systems since the collapsed PMMA cores in the blend system may soften at temperatures above T_g_ of bulk PMMA and start interacting dynamically with PEO chains.

**Figure 6:**
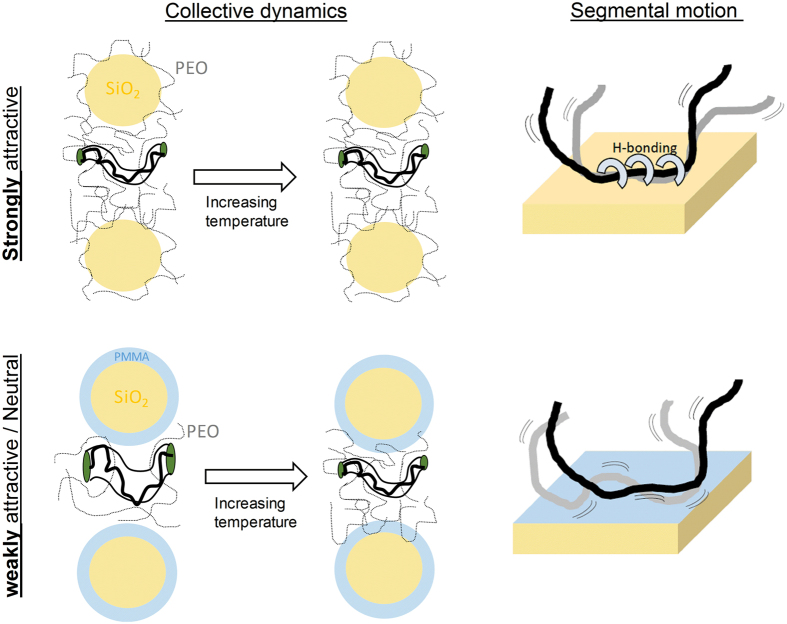
Schematic representation of findings. Tube diameter in strongly attractive composites does not have temperature dependence. Matrix chains disentangle (as depicted with the left drawing in the bottom) when interfacial polymer is dynamically frozen in weakly attractive system. Tube dilation of PEO within glassy PMMA interfaces is presented from the collective dynamics data. The segmental dynamics is slower due to surface pinned (trains) segments in attractive composites and remains bulk-like in weakly attractive composites.

Our study shows that these discrepancies could be attributed to different dynamic behavior at interphases as represented in Fig. 6. In composites with PMMA-adsorbed particles, PEO chains interact with PMMA on particle surfaces. Due to weak enthalpic interaction of PEO and PMMA, this picture is akin to nanocomposites with neutral interfaces. In this system, regardless of the mobility of PMMA, the Rouse rates are identical to PEO homopolymer, contrary to much slower rates observed in highly attractive bare-particle systems. This suggests that the Rouse dynamics is primarily determined by the polymer-surface interaction and remains hardly affected in neutral/weakly attractive composites at interparticle separations greater than the tube size. More importantly, the present work provides an experimental observation of tube dilation in polymer nanocomposites with inorganic nanoparticles interacting weakly with the matrix polymer. The effects of geometric confinement induced by the nanoparticles and chain disentanglements in the presence of well-dispersed weakly attractive interfaces is likely to be the cause of reinforcement or reduced viscosity in many composite systems^49^,^51^,^52^,^53^,^54^. The decrease and recovery of the entanglements by vitrification and softening of the bound polymer aligns with the unusual softening-stiffening transition of our bulk composites and highlights the importance of the interface softness. The microscopic behavior of nanocomposites with dynamically asymmetric polymer blend interphases is clearly different from that of previously studied composite systems. These findings are the first step towards engineering interphases in nanocomposites for new soft active materials.

## Methods

### Materials

Colloidal SiO_2_ nanoparticles (MEK-STL, 55-nm) dispersed in methyl ethyl ketone were supplied by Nissan Chemical America Corporation and used as received. d-PEO (M_n_: 35 kg/mol, Đ:1.09), h-PEO (M_n_: 35 kg/mol, Đ:1.08) and d-PMMA (M_n_: 55 kg/mol, Đ:1.13) were purchased from Polymer Source Inc. h-PMMA (M_n_: 55 kg/mol, Đ:1.13) was purchased from Sigma Aldrich.

### Preparation of Nanocomposites

PMMA adsorption on nanoparticles and nanocomposite film preparation was described elsewhere^25^,^55^. Briefly, PEO with correct D/H ratio was first dissolved at 30 mg/ml in acetonitrile by mechanically stirring the solution at room temperature. The polymer solutions were filtered using a 0.45 *μ*m PTFE filter prior to using in further steps. For the bare SiO_2_-PEO composites, nanoparticles were added into PEO solution and bath-sonicated for 15 min. For the PMMA-adsorbed SiO_2_ composites, polymer-nanoparticle solutions were stirred for 15 min and then poured into PTFE cups. The solvent was evaporated overnight under a fume hood to obtain dry films of 0.2 mm thick at room temperature. Lastly, the nanocomposite films were annealed under vacuum at 90 °C for 2 d to ensure removal of any residual solvent.

### Structural characterization

SEM images were obtained using a Zeiss Auriga Dual-Beam FIB-SEM. Sample films were freeze-fractured in liquid nitrogen. Cross-section images were obtained at 2 kV beam and ≈5 mm working distance at room temperature.

### Rheology

Rheology experiments were conducted on a strain-controlled ARES-G2 rheometer (TA Instruments) equipped with 8-mm parallel plates. The measurements were performed in the linear viscoelastic regime at 353 K and 423 K (below and above the *T*_g_ of PMMA) under nitrogen environment.

### Small-angle neutron scattering (SANS)

SANS was performed on NGB30 at the NIST Center for Neutron Research (NCNR, Gaithersburg, MD). Samples sandwiched between quartz windows were molten at 363 K for 15 min and then equilibrated at 353 K for SANS measurements. The measurements were performed at four configurations for the BS samples with sample-to-detector distances of (1, 4 and 13 m) to cover *Q*-range from ≈0.004 Å^−1^ to 0.5 Å^−1^. A neutron wavelength of λ = 6 Å was used for (1, 4 and 13 m) detector position while λ = 8.4 Å was used for 13 m with lens. Scattering profiles for NSE samples were obtained at the same configurations except that no lens configuration was used. All scattering profiles were corrected for background, empty cell and sample transmission to get 1-D isotropic scattering patterns using Igor based SANS packages^56^ developed by NCNR.

### Quasielastic Neutron Scattering (QENS)

In a dynamic neutron scattering experiment, the double differential scattering cross-section, *i.e.* probability that neutrons are scattered into a solid angle with an energy change, is measured and related to the dynamic structure factors, *S*(*Q*, *ω*), by


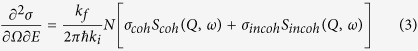


where *σ*_*coh*_ and *σ*_*incoh*_ are the coherent and incoherent atomic scattering cross-sections, respectively, and *N* is the number of atoms. The coherent and incoherent dynamic structure factors


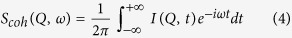



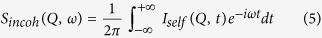


are related to the Fourier-transformed coherent and incoherent (self) intermediate scattering functions by


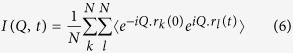



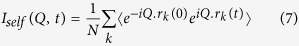


Incoherent scattering is related to the spatial correlations of the same atom at different times and gives the self-motion, while coherent scattering is due to spatial correlations between different atoms at different times and gives the collective motion.

We use fully hydrogenated PEO and fully deuterated PMMA for backscattering experiments to obtain *S*_*incoh*_(*Q*, *ω*) which can be related to the mean-square displacement of the polymer segments from BS. Due to the large incoherent scattering cross-section of H (σ^H-incoh^ = 80 barns) compared to coherent and incoherent cross-sections of all other atoms within the composites (H (σ^H-coh^ = 1.8 barns), D (σ^D-coh^ = 5.6 barns, σ^D-incoh^ = 2 barns), O (σ^O-coh^ = 4.24 barn, σ^O-incoh^ = 0 barns), C (σ^C-coh^ = 5.56 barns, σ^C-incoh^ = 0 barn), Si (σ^Si-coh^ = 2.12 barns, σ^Si-incoh^ = 0 barn)), quasi-elastic neutron scattering (QENS) data are dominated by the self-motion of H atoms through energy change of the scattered neutrons at time scales ranging from 100 ps to ≈2 ns and a *Q*-range relevant to segmental motions.

QENS experiments were performed at NG2-high-flux backscattering spectrometer (HFBS)^57^ at NIST Center for Neutron Research (NCNR). Resolution for each sample was first obtained at 30 K and the samples were heated to desired temperatures at a rate of 3 K/min followed by 1 h thermal equilibration. Doppler shifted neutrons with incident wavelength of 6.27 Å provide a dynamic range of 11 μeV (with resolution of 0.8 μeV). We used a spatial range of (0.25 to 1.75) Å^−1^. Samples were melted on Al foils at 90 ^o^C and pressed using 90 μm spacers to obtain samples with ≈90% transmission. The final films were folded into an annular shape of 3 cm diameter and 3 cm height and placed into an Al can sealed in He environment. An empty cell was used to subtract the background scattering and the detector efficiency was corrected using Vanadium data. The data was reduced and analyzed using the DAVE software^58^.

### Neutron-spin echo (NSE)

In NSE, the intermediate scattering function *S*(*Q*, *t*) is directly measured using the precession of the neutron spin in a magnetic field. The intensity decay is related to the collective PEO dynamics using contrast matched composites. Collective PEO dynamics was obtained using NGA Neutron Spin Echo Spectrometer at NIST Center for Neutron Research (NCNR, Gaithersburg MD). The measurements were performed at 353 K and 423 K for PEO and PEO-PMMA-adsorbed Silica composites; and 373 K and 443 K for PEO-bare Silica composites using wavelength of λ = 11 Å (Δλ/λ = 0.15) for Fourier times up to 100 ns and a spatial range of *Q* = (0.08 to 0.2) Å^−1^. Samples were sealed in Al-cans in a He environment. A standard charcoal sample was used to obtain the instrumental resolution. Data is reduced using the DAVE reduction tool^58^ developed in NCNR.

### Disclaimer

Certain trade names and company products are identified in order to specify adequately the experimental procedure. In no case does such identification imply recommendation or endorsement by the National Institute of Standards and Technology, nor does it imply that the products are necessarily the best for the purpose.

## Additional Information

**How to cite this article**: Senses, E. *et al*. Microscopic Chain Motion in Polymer Nanocomposites with Dynamically Asymmetric Interphases. *Sci. Rep.* **6**, 29326; doi: 10.1038/srep29326 (2016).

## Supplementary Material

Supplementary Information
