# Supplementary material for: Microscopic Chain Motion in Polymer Nanocomposites with Dynamically Asymmetric Interphases
Source: Sci Rep. 2016 Jul 26;6:29326. doi: 10.1038/srep29326 (PMC4960532; doi:10.1038/srep29326)
Supplement: Supplementary Information [file srep29326-s1.pdf]

Supporting information for

Microscopic Chain Motion in Nanocomposites with Dynamically Asymmetric Interphases

Erkan Senses[1, 2,*], Antonio Faraone[1], and Pinar Akcora[3,*]

[1]NIST Center for Neutron Research, National Institute of Standards and Technology

Gaithersburg, MD 20899-8562

[2]Department of Materials Science and Engineering, University of Maryland, College Park, Maryland 20742-2115

[3]Department of Chemical Engineering and Materials Science, Stevens Institute of Technology, Castle Point on Hudson, Hoboken, New Jersey 07030-5942

*Address correspondence to: Erkan Senses (erkan.senses@nist.gov); Pinar Akcora (pinar.akcora@stevens.edu)

Supplementary Table 1. Sample characteristics used in NSE and BS experiments

Experiments	Sample name	D/H (PEO)	D/H (PMMA)	Silica wt.%	Silica vol%
Spin-echo	PEO	0.51/0.49	0.44/0.56	0	0
	PEO-Silica/bare	0.51/0.49	0.44/0.56	30	17.6
	PEO-Silica/PMMA	0.51/0.49	0.44/0.56	30	17.6
Back-scattering	PEO	0/1	1/0	0	0
	PEO-Silica/bare	0/1	1/0	31	18.3
	PEO-Silica/PMMA	0/1	1/0	33	19.7

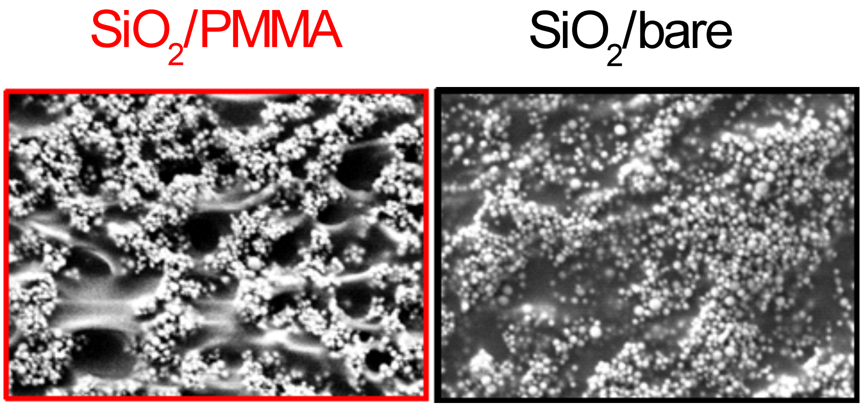

Supplementary Figure 1. Particle aggregates in PEO with hydrophobic ends. Scanning electron micrographs showing poor dispersion of PMMA adsorbing (upper image) and bare (lower image) silica particles in PEO chains with –CH_3 ends compared to individual particle dispersion in OH- terminated hydrophilic PEO.

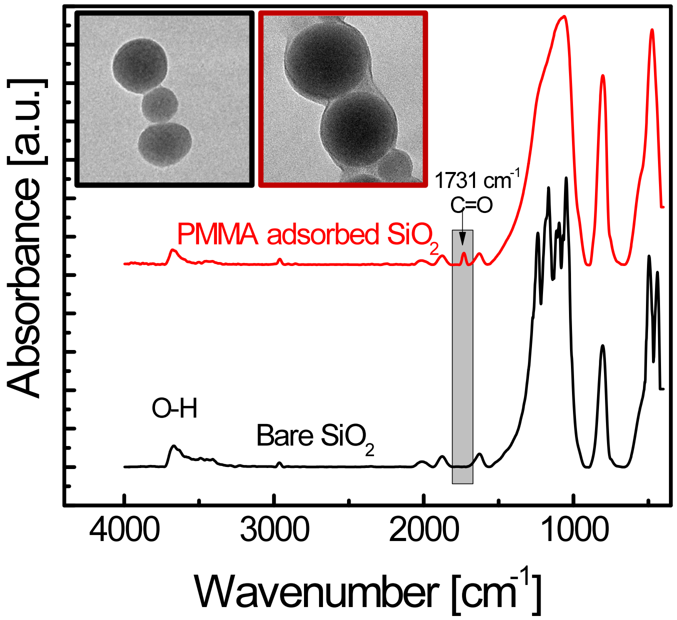

Supplementary Figure 2. IR spectra from bare and PMMA coated particles. FTIR spectra showing C=O vibration of PMMA on silica particles. The insets shows the TEM images of the bare and PMMA adsorbing particles drop cast dilute solutions. The PMMA layer is ≈ 2nm thick.

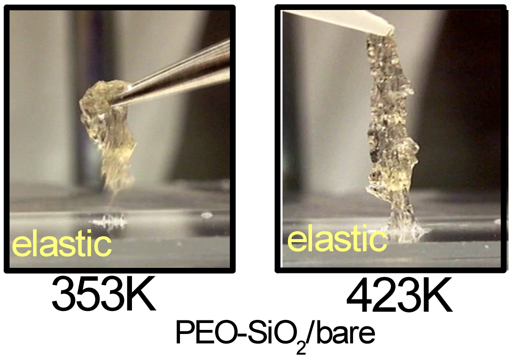

Supplementary Figure 3. PEO nanocomposite with bare SiO_2 particles. The composite with the bare particles and PEO behaves elastic at 353K and 423K.

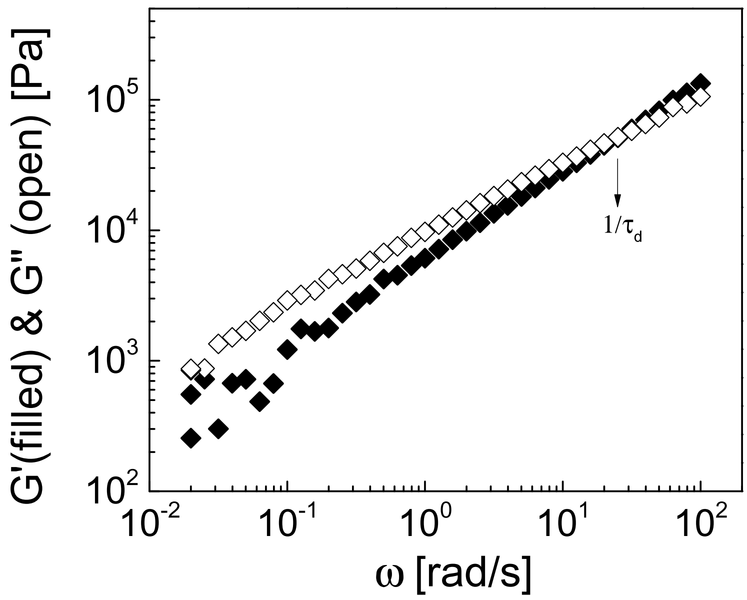

Supplementary Figure 4. Transition from reptation to terminal flow regime in 100 kg/mol PEO homopolymer. Frequency sweep of 100 kg/mol PEO at 348K showing elastic (G') and viscous (G'') moduli and their cross-over related to the terminal relaxation time.

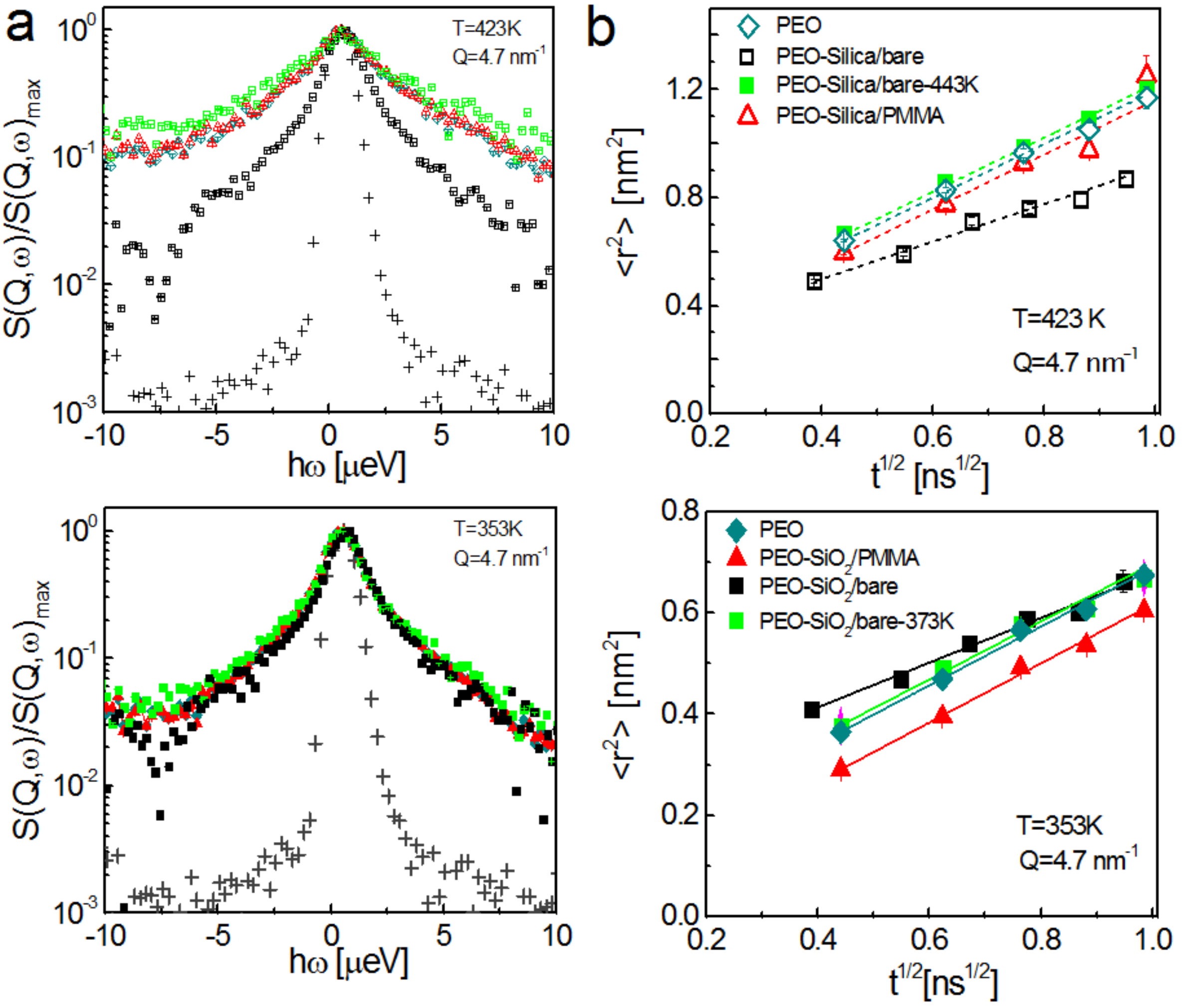

Supplementary Figure 5. (a) Dynamic structure factor of PEO and the nanocomposites in the frequency domain obtained in backscattering at 423K and 353K at Q= 4.67 nm^{-1} **(b).** Mean-square displacement (MSD) obtained from the inverse-Fourier transformed backscattering data at 423K and 353K plotted on Rouse scaling ($\sqrt{t}$).

Neutron Spin-Echo Spectroscopy

It is noteworthy that PMMA is dynamically frozen on the NSE timescale as the temperature is close to its glass transition temperature. Brodeck et al.[26] reported the single chain dynamic structure factor for 23 kg/mol d-PMMA in blends with 25% 23kg/mol h-PEO at 400K as $[S(Q,t)/S(Q,0)]_{PMMA} \approx 1$.Only for the blends of very short chains (≈2 kg/mol, 20:80 PEO/PMMA ratio) the PMMA affected the PEO dynamics; yet the effect was found to be due to lower T_g of short PMMA, a case not relevant in this work. In an independent study, we also measured collective PMMA dynamics in the absence of PEO and found no noticeable relaxation of PMMA even at a temperature ≈80K above the bulk glass-transition temperature of PMMA. In addition, d-PMMA/d-PEO ratio in our samples is less than 3%. Therefore, the measured S(Q,t) is entirely due to d-PEO relative to h-PEO.

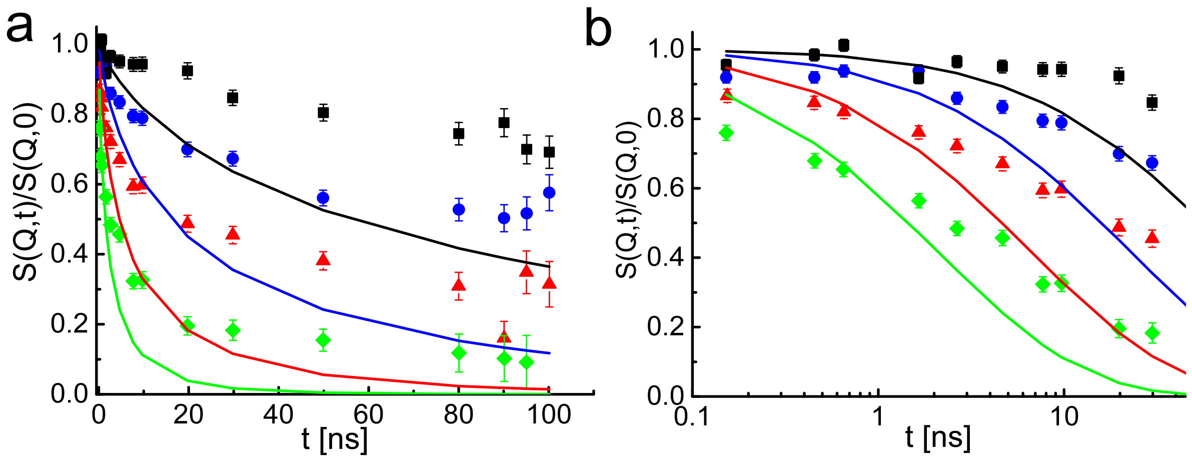

Supplementary Figure 6. Neutron spin-echo results displaying the normalized dynamic structure factors at **(a)** long and **(b)** short times for PEO at 423K. Q values are (0.8, 1.1, 1.5 and 2) nm^{-1} from top to bottom. Solid curves are the predictions from the Rouse model using Rouse rate obtained from the backscattering ($W_{PEO-423K,BS}$).

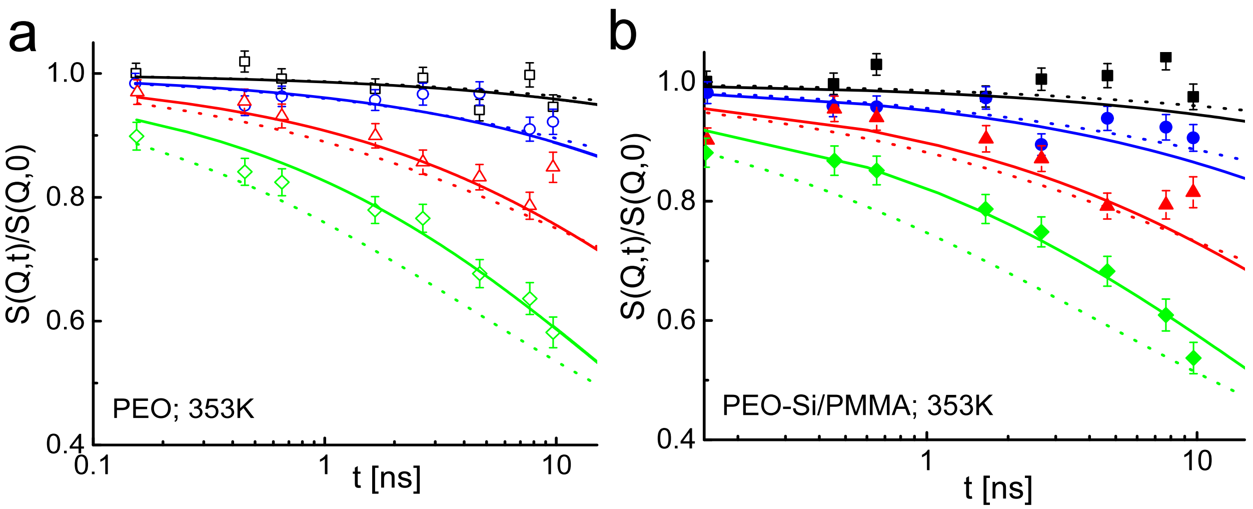

Supplementary Figure 7. Neutron spin-echo results displaying the normalized dynamic structure factors for **(a)** PEO **(b)** PEO in nanocomposite with PMMA coated particle at 353K. Q values are (0.8, 1.1, 1.5 and 2) nm^{-1} from top to bottom. Dashed and solid curves are the predictions from the de Gennes' model using Rouse rate obtained from the backscattering and NSE, respectively.

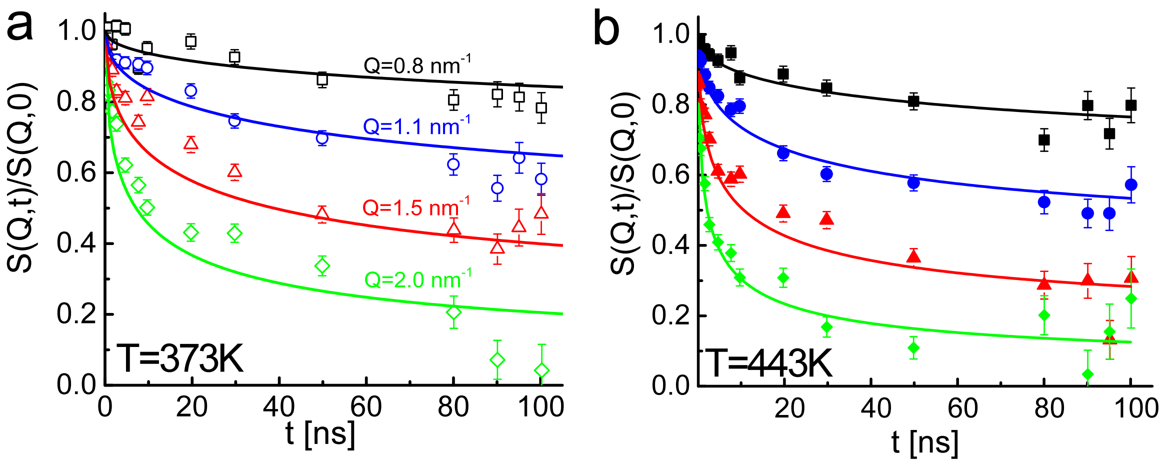

Supplementary Figure 8. Neutron spin-echo results displaying the normalized dynamic structure factor of the composite with bare SiO_2 particles in PEO and corresponding fits to the reptation model at **(a)** 373K and **(b)** 443K.

Supplementary Table 2. Rouse rates obtained from neutron backscattering and spin echo (written italic)

**Sample**	Wl^4 **[nm]**
PEO (T=353K)	0.24 ± 0.008
PEO (T=353K)-NSE	*0.08 ± 0.01*
PEO-Silica/PMMA (T=353K)	0.25 ± 0.014
PEO-Silica-bare (T=353K)	0.15 ± 0.008
PEO-Silica-bare (T=373K)	0.26 ± 0.012
PEO (T=423K)	0.72 ± 0.025
PEO (T=423K)-NSE	*0.56 ± 0.06*
PEO-Silica/PMMA (T=423K)	0.73 ± 0.042
PEO-Silica-bare (T=423K)	0.40 ± 0.022
PEO-Silica-bare (T=443K)	0.78 ± 0.014

Supplementary Table 3. Tube diameter of PEO obtained from neutron-spin echo data

Sample	*d* [nm]
PEO (T=353K)	5.57 ± 0.4
PEO (T=423K)	5.56 ± 0.2
PEO-Silica/PMMA (T=353K)	6.78 ± 0.3
PEO-Silica/PMMA (T=423K)	5.49 ± 0.1
PEO-Silica-bare (T=373K)	5.24 ± 0.2
PEO-Silica-bare (T=443K)	5.63 ± 0.2

References

1 Fetters, L., Lohse, D. & Colby, R. in *Physical Properties of Polymers Handbook* 447-454 (Springer, 2007).

2 Senses, E., Isherwood, A. & Akcora, P. Reversible Thermal Stiffening in Polymer Nanocomposites. *ACS Applied Materials & Interfaces* **7**, 14682-14689 (2015).

3 Senses, E. & Akcora, P. Tuning mechanical properties of nanocomposites with bimodal polymer bound layers. *RSC Advances* **4**, 49628-49634 (2014).

4 Kline, S. Reduction and analysis of SANS and USANS data using IGOR Pro. *Journal of Applied Crystallography* **39**, 895-900 (2006).

5 Azuah, R. T. *et al.* DAVE: a comprehensive software suite for the reduction, visualization, and analysis of low energy neutron spectroscopic data. *Journal of Research of the National Institute of Standards and Technology* **114**, 341-358 (2009).
